# Cultivation of shear stress sensitive and tolerant microalgal species in a tubular photobioreactor equipped with a centrifugal pump

**DOI:** 10.1007/s10811-015-0559-8

**Published:** 2015-03-20

**Authors:** Michiel H. A. Michels, Atze Jan van der Goot, Marian H. Vermuë, René H. Wijffels

**Affiliations:** 1 Bioprocess Engineering, AlgaePARC, Wageningen University, P.O. Box 16, 6700 AA Wageningen, The Netherlands; 2 HZ University of Applied Sciences, P.O. Box 364, 4380 AJ Vlissingen, The Netherlands; 3 Food Process Engineering, Wageningen University, P.O. Box 17, 6700 AA Wageningen, The Netherlands; 4 Faculty of Biosciences and Aquaculture, University of Nordland, 8049 Bodø, Norway

**Keywords:** Shear stress, Sensitivity, Microalgae, Recirculation pump, Tubular photobioreactor

## Abstract

The tolerance to shear stress of *Tetraselmis suecica*, *Isochrysis galbana*, *Skeletonema costatum*, and *Chaetoceros muelleri* was determined in shear cylinders. The shear tolerance of the microalgae species strongly depends on the strain. *I. galbana*, *S. costatum*, and *C. muelleri* exposed to shear stress between 1.2 and 5.4 Pa resulted in severe cell damage. *T. suecica* is not sensitive to stresses up to 80 Pa. The possibility to grow these algae in a tubular photobioreactor (PBR) using a centrifugal pump for recirculation of the algae suspension was studied. The shear stresses imposed on the algae in the circulation tubes and at the pressure side of the pump were 0.57 and 1.82 Pa, respectively. The shear stress tolerant *T. suecica* was successfully cultivated in the PBR. Growth of *I. galbana*, *S. costatum*, and *C. muelleri* in the tubular PBR was not observed, not even at the lowest pumping speed. For the latter shear sensitive strains, the encountered shear stress levels were in the order of magnitude of the determined maximum shear tolerance of the algae. An equation was used to simulate the effect of possible damage of microalgae caused by passages through local high shear zones in centrifugal pumps on the total algae culture in the PBR. This simulation shows that a culture of shear stress sensitive species is bound to collapse after only limited number of passages, confirming the importance of considering shear stress as a process parameter in future design of closed PBRs for microalgal cultivation.

## Introduction

*Skeletonema*, *Chaetoceros*, *Phaeodactylum*, *Isochrysis*, *Pavlova*, and *Tetraselmis* are frequently used microalgae in feed applications for shellfish in hatcheries (Muller-Feuga 2013). These species would be candidates for large scale production given the increasing demand for microalgal feed of constant and high quality for aquaculture. An increased level of microalgae (components) in fish feed is interesting for the development of fish feed with reduced environmental impact (Draganovic et al. 2013).

For controlled large-scale cultivation of microalgae, closed photobioreactors (PBRs) are often advocated (Borowitzka 1999; Acién et al. 2012). Closed PBRs were combine with low contamination risk and almost no CO_2_ losses leading to a high productivity. Furthermore, the cultivation can be fully automated and culture conditions can be highly controlled (Pulz 2001). However, hydrodynamic forces evoked by turbulent mixing in closed PBRs may limit the application of these closed systems. These forces must not exceed the level that would lead to detrimental effects (Chisti 2010; Tredici 2010), suggesting the importance of limiting the shear stress in such PBR systems to levels that can be tolerated by the microalgae. This is especially important when considering the fact that digestibility of microalgael species is positively related to the fragility of the cells (Gladue and Maxey 1994) and therefore to the way many microalgal species used in aquaculture can be cultivated.

Table 1 summarizes the culture systems being employed for the cultivation of the most frequently used microalgal species for aquaculture. All these species can successfully be grown in bubble columns, bags, and carboys; systems that use aeration for mixing. Other types of PBRs, like horizontal tubular PBRs with a shorter light path to produce microalgae at a higher density, are used by aquaculture facilities more often lately (Zmora et al. 2013). Mixing in these systems is induced through recirculation of the microalgal culture in the tubular PBR using pumps, of which centrifugal pumps and airlift pumps are the most common ones (Alías et al. 2004; Molina et al. 2001). Centrifugal pumps are efficient in gas–liquid mass transfer (Fadavi and Chisti 2005) and energy use (Norsker et al. 2011). However, microalgal productivity can be influenced negatively by using centrifugal pumps, due to cell damage occurring inside the pump (Carvalho et al. 2006). This could be an explanation for the very few records of microalgae species used in aquaculture, which have been successfully cultivated in tubular PBRs with centrifugal pumps (Table 1). It is still not clear if shear stress sensitivity of certain microalgal species enables successful cultivation in closed PBRs. Although shear stress sensitive species are being cultivated in tubular PBRs with airlift pumps, shear stress levels occurring in the tubes of PBRs could already be too high resulting in loss of productivity. Therefore, there is a need to study the relationship between shear stress sensitivity of microalgal strains and shear stress levels encountered in closed PBRs.

**Table 1:** Most frequently used microalgal species as feed for larvae of mollusks, shrimp, and live prey for fish larvae and their culture systems

Class	Species	Culture system	Mixing	References
Bacillariophyceae	*Skeletonema costatum*	Raceway	Air	Hussenot et al. (1998)
		Polyethylene bags	Air	Pronker et al. (2015)
		Airlift PBR, bubble column	Airlift, air	Monkonsit et al. (2011)
	*Chaetoceros muelleri, Chaetoceros gracilis, Chaetoceros calcitrans*	Polycarbonate carboys	Air	Camus and Zeng (2012)
		Polyethylene bags	Air	Kaspar et al. (2014); Pronker et al. (2015)
		Bubble column	Air	Lee et al. (2011)
		Airlift PBR, bubble column	Airlift, air	Krichnavaruk et al. (2005)
	*Phaeodactylum tricornutum*	Polyethylene bags	Air	Pronker et al. (2015)
		Bubble column	Air	Lee et al. (2011)
		Tubular PBR	Airlift	Acién Fernández et al. (2000)
		Tubular PBR	Centrifugal pump	Silva Benavides et al. (2013)
Prymnesiophyceae	*Isochrysis galbana*	Polycarbonate carboys	Air	Camus and Zeng (2012)
		Polyethylene bags	Air	Dunstan et al. (1993); Kaspar et al. (2014); Pronker et al. (2015)
		Bubble column	Air	Lee et al. (2011)
		Airlift PBR	Airlift	Loubière et al. (2009)
		Tubular PBR	Airlift	Molina Grima et al. (1994); Van Bergeijk et al. (2010)
		Tubular PBR	Centrifugal pump	Van Bergeijk et al. (2010)
	*Pavlova lutheri,* *Pavlova salina*	Polycarbonate carboys	Air	Camus and Zeng (2012)
		Polyethylene bags	Air	Dunstan et al. (1993); Pronker et al. (2015)
Prasinophyceae	*Tetraselmis suecica,* *Tetraselmis chuii*	Polycarbonate carboys	Air	Camus and Zeng (2012)
		Polyethylene bags	Air	Moheimani (2013); Pronker et al. (2015)
		Bubble column	Air	Lee et al. (2011)
		Annular column	Air	Chini Zittelli et al. (2006)
		Green wall panel reactor	Air	Bondioli et al. (2012)
		Tubular PBR	Centrifugal pump	Michels et al. (2014)

The aim of the study is to determine the tolerance to shear stress of four different microalgal species. Shear cylinders were used to quantify the threshold values of shear stress for the different microalgal species. These microalgae species were all tested in a tubular PBR with a variable-frequency-drive centrifugal pump to determine the capability of growth. Growth or lack of growth of microalgae will be related to estimated shear stress levels inside the reactor and shear tolerance levels determined for all four species.

## Material and methods

### Organisms and medium

*Tetraselmis suecica*, *Isochrysis galbana*, and *Skeletonema costatum* were obtained from Seasalter Shellfish (Whitsable) Ltd. (Kent, UK), and *Chaetoceros muelleri* (CCMP 1316) was provided by NIOZ (Royal Netherlands Institute for Sea Research). Walne medium modified from Laing (1991) was used for the cultivation of the microalgal species. The modified Walne medium consists of solution A (macro- and micronutrients), C (vitamins), and D (silicate, which is only needed for diatoms). Solution A contains 0.8 g FeCl_3_, 0.4 g MnCl_2_ · 4H_2_O, 33.6 g H_3_BO_3_, 45.0 g EDTA, 20.0 g NaH_2_PO_4_·2H_2_O, 100.0 g NaNO_3_, 21 mg ZnCl_2_, 20 mg CoCl_2_·6H_2_O, 9.0 mg (NH_4_)_6_Mo_7_O_24_·4H_2_O, and 20 mg CuSO_4_·5H_2_O in 1 L of distilled water, with the pH adjusted to 4.0 with concentrated HCl. Solution C consists of 1.0 g vitamin B_1_ and 0.05 g vitamin B_12_ in 1 L of distilled water. Solution D contains 40.0 g Na_2_SiO_3_·5H_2_O in 1 L of distilled water. Medium for maintaining the cultures in Erlenmeyer flasks was made by adding 1 mL solution A, 0.1 mL solution C, and 2 mL solution D per liter of filtered and deironized saline groundwater (30 g L^−1^). A double dose of solutions A, C, and D was supplied to the culture in the tubular photobioreactor during turbidostat operation to avoid nutrient depletion. Solution D was only added for the cultivation of the diatoms *S. costatum* and *C. muelleri*.

### Generation of shear stress levels tolerance

Batch cultures of *T. suecica*, *I. galbana*, and *S. costatum* from 3-L Erlenmeyer flasks containing 2 L medium after 1 week of growth at 20 °C under white fluorescent light (150 μmol photons m^−2^ s^−1^; PAR) were used for shear stress experiments. The three microalgal strains were exposed to different shear stress levels in shear cylinders as Couette devices (Van Riemsdijk et al. 2010). Shear stress exposed to the microalgae can be determined with:

$$ \tau =\dot{\gamma}\cdot \eta $$

where *τ* is the shear stress (Pa), $\dot{\gamma}$ is the shear rate (s^−1^), and *η* is the apparent viscosity (Pa s). Shear stress levels can be varied by increasing the shear rate or by increasing the apparent viscosity of the medium. The shear rate applied in the shear cylinders is related to the rotational speed with a conversion factor of rotational speed (rpm) to shear rate (s^−1^) of 2.157 (Michels et al. 2010). Locust bean gum (LBG), which does not affect the viability directly (Michels et al. 2010), was used as a thickener to increase the apparent viscosity.

*S. costatum* and *I. galbana* with 0.3 % LBG in the medium were exposed to different shear rates of 0, 8.6, 43, 216, and 1079 s^−1^. *T. suecica* also was exposed to the same shear rates, but with two LBG concentrations: 0.5 and 0.75 % LBG, to obtain higher viscosity. All exposures were done in triplicate at 4 °C with an exposure time of 1 h. Since LBG solutions can be described as non-Newtonian fluids, the apparent viscosity was measured at the different shear rates in order to calculate the applied shear stress levels. The shear stress levels applied in the shear cylinders were determined with a rheometer (type Physica MCR 301, Anton Paar) at 4 °C according to the method described in Michels et al. (2010). The power-law functions that describe the relation between shear rate and shear stress in 0.3, 0.5, and 0.75 % LBG are $\tau =0.034{\dot{\gamma}}^{0.943}$ with *R* ^2^ = 0.9998, $\tau =0.227{\dot{\gamma}}^{0.763}$ with *R* ^2^ = 0.993 and $\tau =1.175{\dot{\gamma}}^{0.618}$ with *R* ^2^ = 0.987, respectively. These functions were used to calculate the shear stress levels applied at different rotational speed levels and LBG concentrations. Table 2 presents the calculated shear stress levels related to rotational speed, shear rate, and LBG concentration.

**Table 2:** Shear stress applied in relation with rotational speed, shear rate, and LBG concentration

Rotational speed (rpm)	Shear rate (s^−1^)	Shear stress 0.3 % LBG (Pa)	Shear stress 0.5 % LBG (Pa)	Shear stress 0.75 % LBG (Pa)
0	0	0	0	0
4	8.6	0.26	1.2	4.5
20	43	1.2	4.0	12
100	216	5.4	14	33
500	1079	25	47	88

### Measurement of effect of shear stress on viability

The effect of applying shear stress on the viability of *T. suecica*, *S. costatum*, and *I. galbana* was measured by using fluorescein diacetate (FDA). Viable cells contain esterases that convert FDA into fluorescein and diacetate. Viable cells show fluorescence caused by fluorescein (Altman et al. 1993; Rotman and Papermaster 1966). After exposure in the shear cylinders, 1 mL of algae was incubated with 10 μL FDA solution (11 mM) for 20 min. The total cell concentration and viable cell concentration were determined with a hemocytometer (DHC-B02-5 Büker Türk) using a fluorescence microscope. The viability of the sheared algae was calculated as the percentage of fluorescing algae and compared to the shear stress tolerance of *C. muelleri* (Michels et al. 2010).

Because *S. costatum* can form chains up to 8–10 cells per chain, the effect of shear stress on the distribution in the number of cells per chain was also measured. The number of cells per chain was counted for at least 50 chains per sample. The Mann–Whitney *U* test was done to determine any statistical differences in cells per chain distribution per applied shear stress.

### Growth of microalgae in a tubular photobioreactor

Growth tests with *T. suecica*, *I. galbana*, *S. costatum*, and *C. muelleri* were carried out in a tubular PBR with a total volume of 40 L. The tubular PBR consists of 20-m-long loop connected to a degasser. The loop is made of Plexiglas tubes with an external diameter of 50 mm and an internal diameter 43.6 mm. This culture system is equipped with a variable-frequency-drive centrifugal pump (SealPro KR-32-95, ARBO) with an impeller diameter of 95 mm and a pressure side diameter of 32 mm, to circulate the microalgal culture. The location and main operating mode of the tubular PBR are described in Michels et al. (2014).

The four microalgal species were separately used for inoculation of the tubular PBR with a minimal starting cell concentration of 200,000 cells mL^−1^. Temperature was controlled at 20 ± 0.5 °C. The pH is measured at the end of the tube before the degasser and controlled at pH 8.40 via CO_2_ supply. The initial pumping speed at which the microalgae were recirculated was 0.37 m s^−1^, with a rotational speed of the impeller of 10 s^−1^.

The increase in cell concentration was followed by taking daily samples for 7 days. The run in the PBR was terminated when the cell concentration of an algae species did not increase during this period. The PBR was cleaned thoroughly and inoculated with the same species for a second time. The daily samples were inspected with a microscope to check the shape of the microalgae cells, the motility of the flagellates and potential bacterial occurrence.

The runs in which the algae concentration increased during the first 7 days were further used to study the effect of pumping speed on the net volumetric productivity at turbidostat conditions, with the biomass concentration set at about 0.5 g L^−1^. The pumping speed of the recirculation pump was 2.0, 2.4, 2.8, 3.2, and 3.6 m^3^ h^−1^, respectively. Those trials were done for a period of 14 days.

### Calculation of shear stress levels in the tubular photobioreactor

The corresponding flow velocities and Reynolds numbers in the tubes and at the pressure side of the pump were calculated, with a known culture density of 1024 kg m^−3^ and an apparent viscosity of 1.8 · 10^−3^ Pa s (Michels et al. 2010). Those conditions were used to estimate the average shear stress levels in the tubes. The Blasius equation was used to calculate the shear stress at the wall of the tube where flow will be laminar (Durst et al. 1996). The equation is:

$$ \tau ={C}_{\mathrm{f}}\frac{1}{2}\rho {\overline{u}}^2 $$

with *C*_f_ = 0.0791*Re* ^‐ 1/4^

where *τ* is the average shear stress (Pa), *C*_f_ is the Fanning friction factor (dimensionless), *ρ* is the density (kg m^−3^), *ū* is the average flow velocity (m s^−1^), and *Re* is the Reynolds number. The Reynolds number is expressed by:

$$ Re=\frac{\rho \cdot \overline{u}\cdot D}{\eta } $$

where *D* is the internal diameter (m).

The shear stress in the centrifugal pump depends on the apparent viscosity, rotational speed of the impeller, and the Reynolds number. The local shear stress adjacent to the rotating impeller of the centrifugal pump can be estimated with:

$$ \tau =6.30\eta NR{e}_L^{0.5} $$

where *N* is the rotational speed of the impeller and the local Reynolds number at the local diameter is defined as:

$$ R{e}_{\mathrm{L}}=\frac{N{d}_{\mathrm{L}}^2\rho }{\eta } $$

where *d*_L_ is the local diameter of the impeller (Chisti 2010; Wichterle et al. 1996).

### Determination of the net volumetric productivity

The net volumetric productivity (*P*_V_, g L^−1^ day^−1^) was determined as the product of the net-specific growth rate (μ, d^−1^) and the biomass concentration (*C*_X_, g L^−1^). At turbidostat conditions, the net-specific growth rate is equal to the dilution rate (D, d^−1^), defined as the daily harvest volume divided by the volume of the PBR (Michels et al. 2014).

The daily photon fluxes were derived from the measured photosynthetically active radiation (PAR) as an average daily incident photon flux density (PFD, mol photons m^−2^ day^−1^) (Michels et al. 2014). The PFD was measured with a LiCor LI190 PAR sensor.

The average daily photon flux density during culture ranged from 0.69 to 13.3 mol photons m^−2^ day^−1^.

## Results and discussion

### Shear stress tolerance

**Fig. 1:**
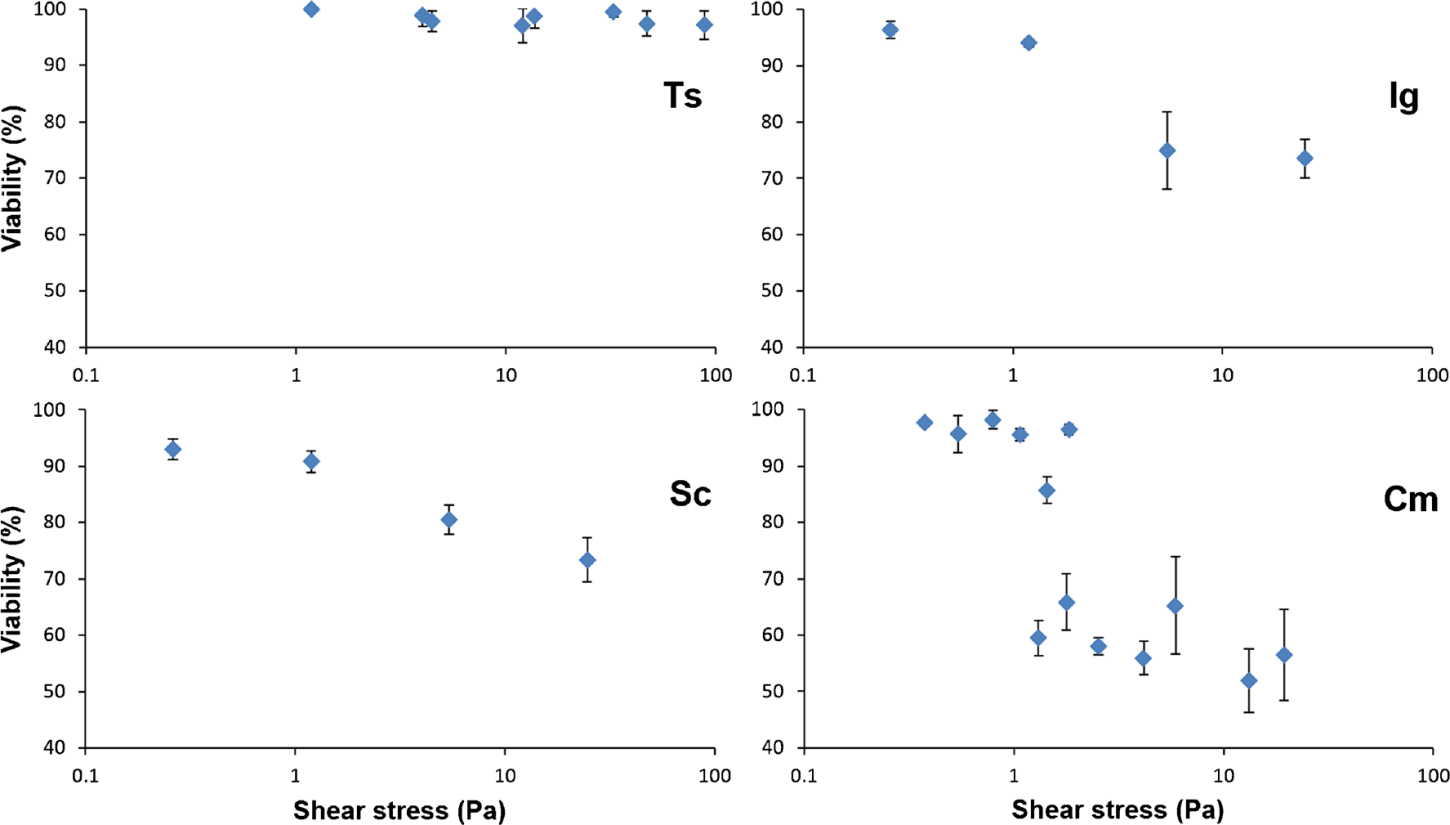
Effect of shear stress on *Tetraselmis suecica* (Ts), *Isochrysis galbana* (Ig), *Skeletonema costatum* (Sc), and *Chaetoceros muelleri* (Cm). Data from effect of shear stress on *Chaetoceros muelleri* were obtained from Michels et al. (2010)

Figure 1 shows the effect of shear stress on the viability of *T. suecica*, *I. galbana*, *S. costatum*, and *C. muelleri*. An adverse effect was found for *I. galbana*, where viability decreased suddenly to 74.9 % evoked by a shear stress level of 5.4 Pa. The viability of *I. galbana* was not reduced any further when the shear stress increased to 25 Pa. *I. galbana* is a flagellate and a member of the non-calcified coccolithophytes with only a plasma membrane covering, which makes the naked cell fragile (Graham et al. 2009b; Zhu and Lee 1997).

**Fig. 2:**
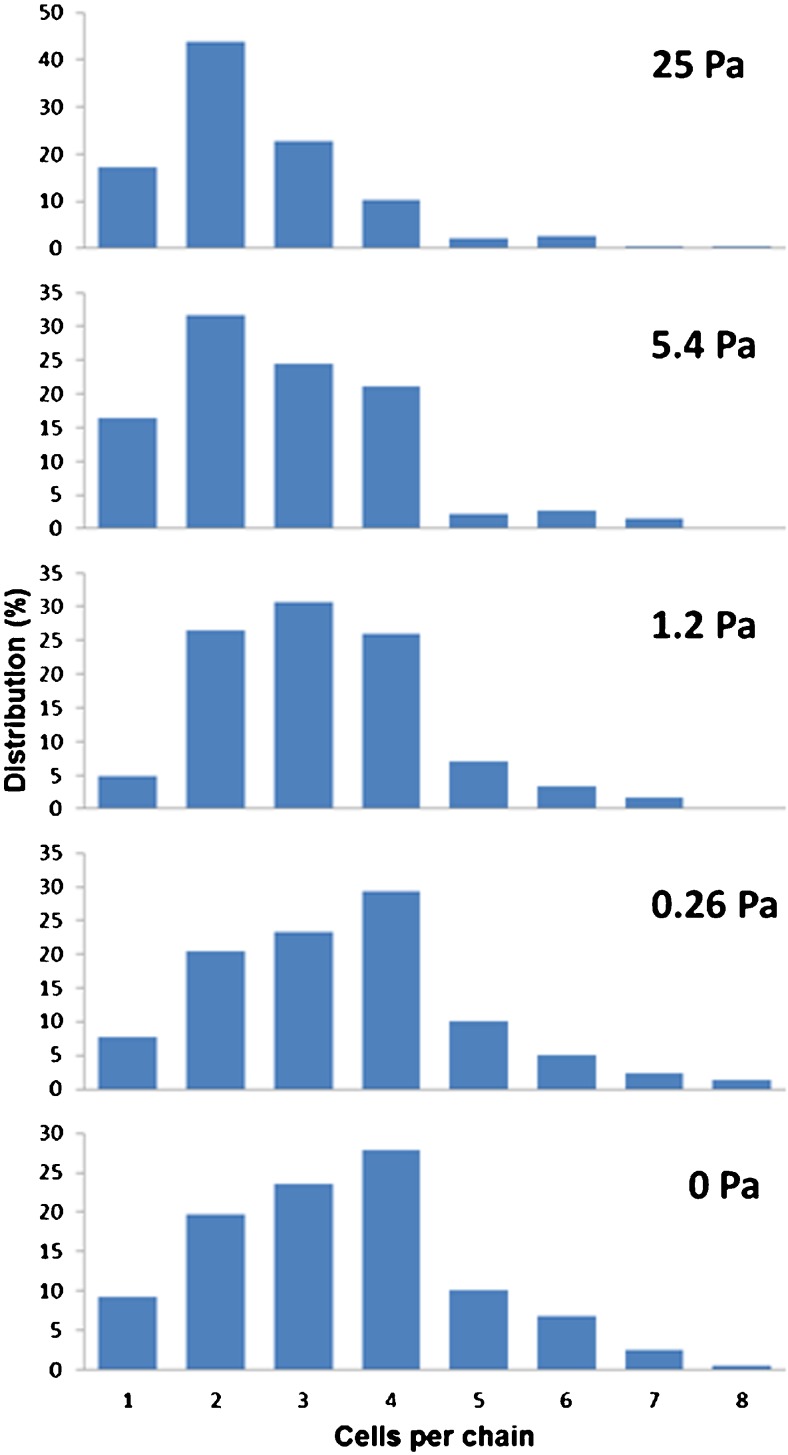
Effect of shear stress on the distribution of cells per chain of *Skeletonema costatum*

The effect of shear stress on *S. costatum* was a bit different. The viability of *S. costatum* decreased further from 80.5 to 73.4 %, when the shear stress increased from 5.4 to 25 Pa. This observation can be ascribed to a change in morphology of *S. costatum* due to the increased shear stress. Since this diatom forms chains, breakages of the chains were expected at higher shear stress levels (Sauriau and Baud 1994). The effect of shear stress on the distribution of cells per chain is shown in Fig. 2. The distribution of cells per chain of unexposed *S. costatum* and cells exposed to shear stress of 0.26 Pa did not differ, while significant differences in distribution of cells per chain were found between all the exposed shear stress levels. Shear stress levels higher than 0.26 Pa caused a significant reduction in the average chain length with a progressive increase of chains with 1–3 cells. The average chain length reduced linearly on a semi-logarithmic scale from 3.46 ± 0.18 to 2.49 ± 0.04 cells per chain with corresponding shear stresses of 0.26 and 25 Pa. Although a shear stress of 1.2 Pa already caused a reduction of the average number of cells per chain, the viability did not decrease significantly (Fig. 1). Shear stress probably has first an impact on the intercellular junctions causing chain breakage, which is then followed by other cell structures like the siliceous frustules being damaged causing mortality (Sauriau and Baud 1994).

Data of *C. muelleri* were used as reference and were obtained from Michels et al. (2010). *C. muelleri* was found to be shear stress sensitive with a threshold value of shear stress between 1 and 1.3 Pa. Higher shear stress levels caused a decrease in viability with a certain fraction of the cells being sensitive to shear stress (Michels et al. 2010).

*T. suecica* exposed to shear stress levels up to 88 Pa did not show any adverse effects on the viability (Fig. 1). The tolerance to high shear stress of *T. suecica* is probably caused by the rigid cell wall composed of layers of scales attached to the cell membrane (Graham et al. 2009a).

For the algae species that were susceptible to shear stress, the viability was decreased with 20 to 40 %. The fact that not all cells were inactivated could be explained by the fact that cells are not equally susceptible to shear damage during the full growing cycle. It has been reported that microalgae are more vulnerable to shear stress during cell division (García Camacho et al. 2007; Stoecker et al. 2006). This will cause a loss of viability of only a certain percentage of the shear stress sensitive species *I. galbana*, *S. costatum*, and *C. muelleri* (Fig. 1). A plausible explanation why the flagellate *T. suecica* is not shear stress sensitive during cell division, is that the flagella are shed during the division prior to mitosis, and cytokinesis takes place within the rigid parental wall (Graham et al. 2009a).

### Growth tests with shear stress sensitive and tolerant microalgal species

**Fig. 3:**
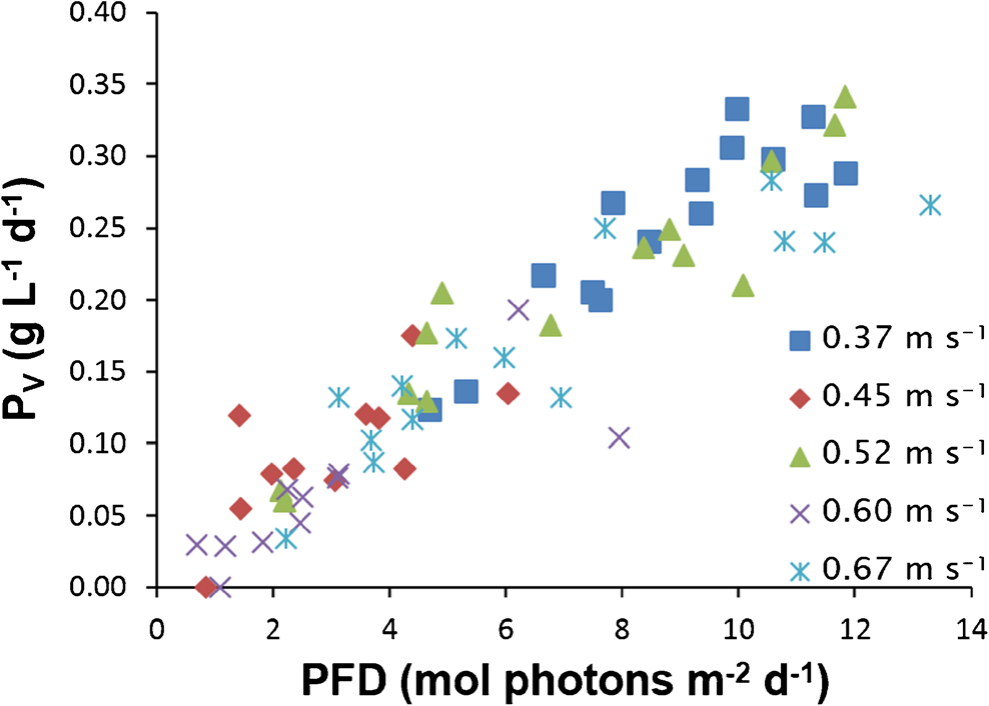
Net volumetric productivity of *Tetraselmis suecica* versus the daily photon flux density at different pumping speeds

*T. suecica*, *I. galbana*, *S. costatum*, and *C. muelleri* were all tested for their capability to grow in a tubular PBR in which the culture is recirculated using a centrifugal pump. *T. suecica* was the only species tested that grew well in the tubular PBR. The net volumetric productivity of *T. suecica* cultivated at different pumping speeds is shown in Fig. 3. The biomass concentration was kept constant at 0.52 ± 0.05 g L^−1^ applying turbidostat conditions. The net volumetric productivity linearly increased with the daily photon flux received. During the entire experiment, the microalgae were exposed to relatively low light intensity. At these low light intensities, no light saturation or light inhibition occurs. A linear increase of productivity with light input is therefore to be expected (Geel et al. 1997).

No differences in net volumetric productivity of *T. suecica* receiving similar light were found between the cultivation periods at different pumping speeds. Higher pumping speed evokes increased shear stress and could affect the net productivity negatively by its potential detrimental effects on the microalgae. On the other hand, it can also have a positive effect on the net volumetric productivity due to a better mass and gas transfer and shorter light/dark cycles that are produced when mixing is increased (Contreras et al. 1998; Leupold et al. 2013; Vejrazka et al. 2012). Neither negative nor positive effects of pumping speed on the productivity of *T. suecica*, however, were found during this study.

The average net volumetric productivities with corresponding average daily photon flux densities for the five runs with a different pumping speed are given in Table 3. During the experimental period at limiting light conditions during the fall, the maximum net volumetric productivity of the microalgae receiving a daily photon flux density of 11.8 mol photons m^−2^ day^−1^ was 0.34 g L^−1^ day^−1^, while daily light inputs lower than 1 mol photons m^−2^ day^−1^ resulted in a zero or close to zero productivity (Fig. 3). The daily photon flux density of 1 mol photons m^−2^ day^−1^ was obviously the compensation point of photosynthesis, at which the rate of photosynthesis equals the respiration rate. The compensation point of photosynthesis of *T. suecica* was similar to the value of *Chlamydomonas reinhardtii* (Takache et al. 2010).

**Table 3:** Average net volumetric productivity and average daily photon flux density at different runs

Pumping speed (m^3^ h^−1^)	Net volumetric productivity (g L^−1^ day^−1^)	Daily photon flux density (mol m^−2^ day^−1^)
2.0	0.25 ± 0.06	8.76 ± 2.17
2.4	0.09 ± 0.05	3.02 ± 1.58
2.8	0.20 ± 0.09	7.14 ± 3.35
3.2	0.07 ± 0.05	2.94 ± 2.23
3.6	0.17 ± 0.08	6.66 ± 3.56

### Biofouling at high pumping speed

Although the highest pumping speed did not lead to a lower net volumetric productivity, the culture of *T. suecica* was affected negatively. Biofouling started to occur in the tubes 1 day after the pumping speed was set at 3.6 m^3^ h^−1^, at which the average shear stress in the tubes and at the pressure side of the pump were 1.60 and 5.10 Pa, respectively (Table 4). Due to the presence of most of the biofouling at the bottom of the tubes, the cells were still able to receive light, which did not result in a lower net volumetric productivity. Biofouling in the tubes increased enormously during the 2 weeks *T. suecica* was cultivated at this high pumping speed. Therefore, *T. suecica* should be cultured at lower pumping speeds to ensure cells will not be damaged. An additional benefit of applying a lower pumping speed is the lower energy costs.

**Table 4:** Flow velocities, Reynolds numbers, and average shear stress levels in tubes and at pressure side of the pump at different pumping speeds

	Tubes			Pressure side of pump		
Pumping speed (m^3^ h^−1^)	Flow velocity (m s^−1^)	Reynolds number	Average shear stress (Pa)	Flow velocity (m s^−1^)	Reynolds number	Average shear stress (Pa)
2.0	0.37	9.2 · 10^3^	0.57	0.69	1.3 · 10^4^	1.82
2.4	0.45	1.1 · 10^4^	0.79	0.83	1.5 · 10^4^	2.51
2.8	0.52	1.3 · 10^4^	1.03	0.97	1.8 · 10^4^	3.29
3.2	0.60	1.5 · 10^4^	1.30	1.11	2.0 · 10^4^	4.15
3.6	0.67	1.7 · 10^4^	1.60	1.24	2.3 · 10^4^	5.10

Growth of recirculated *I. galbana*, *S. costatum*, and *C. muelleri* in the tubular PBR was not observed, not even at the lowest pumping speed of 2.0 m^3^ h^−1^. The cell concentration of these three microalgae species did not increase during the 7 days tested. General observations in chronological order were a decrease of cell concentration within 1 or 2 days, an increase of number of bacteria, followed by foam formation and finally biofouling. In the case of *I. galbana*, the remaining viable cells did not lose their motility and no alteration of the shape could be observed. Regarding *S. costatum*, longer chains disappeared after 1 day with more short chains and single cells as a result. Broken and disintegrated cells of *S. costatum* were seen after a few days. The shape of the remaining cells of *C. muelleri* did not change immediately, but after only a few days the cells became spherical and lost their spines. Shear stress most likely caused the incapability of *I. galbana*, *S. costatum*, and *C. muelleri* to grow in the tubular PBR.

### Relative high shear-stress levels encountered in the photobioreactor

Lack of growth of these microalgae strains could be correlated to the shear stress sensitivity of the cells investigated. Unfortunately, exact shear stress values are difficult to predict in turbulent flow, but the stress at the wall can be calculated easily, due to the fact that flow is laminar at the wall. The shear stress at the wall is 0.57 Pa at a pumping speed of 2.0 m^3^ h^−1^. A similar calculation leads to a wall shear stress of 1.82 Pa in the pressure side of the pump (Table 4). It can be expected that even higher shear stress values can occur in other parts of the reactor. For example, maximum shear stress levels of about 2 Pa were reported in bends of tubes with similar diameters (5 cm) and at similar velocity (0.35 m s^−1^) in the tubes based on computational fluid dynamics (Ramírez-Duque and Ramos-Lucumi 2011). Even higher shear stress levels will occur in the cavity of the centrifugal pump, where the shear forces are not equally distributed. With an impeller of 95 mm and rotational speed of 10 s^−1^, the maximum shear stress occurring in the centrifugal pump could be calculated. The maximum shear stress at the tip of the impeller in the centrifugal pump at a pumping speed of 2.0 m^3^ h^−1^ is 26 Pa. Comparing this shear stress level with the shear stress that evokes loss of viability of the cells (Fig. 1), it is obvious that local shear stress in the pump must have been detrimental to the shear stress sensitive microalgae species. However, a large part of the reactor might still have favorable conditions for cell cultivation.

### Simulation of the possible damage caused by high circulation rates

**Fig 4:**
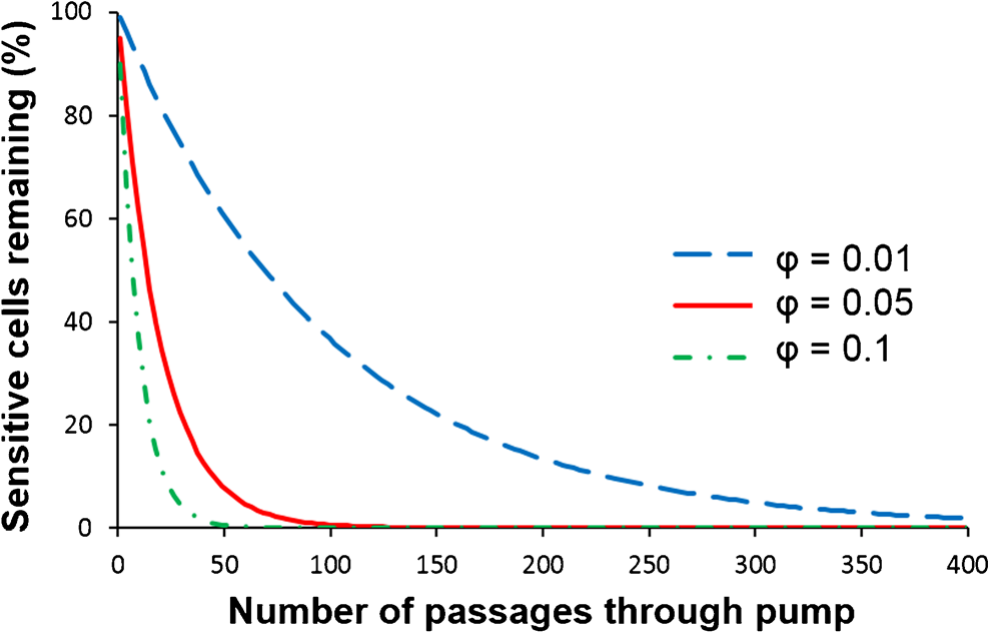
Percentage of remaining cells as an effect of passages through a pump and proportion of cells being damaged per passage

Unfortunately, the high circulation rate of the culture will inevitably lead to the situation that all cells will pass a high shear zone and most of them will pass the zone several times. This can be demonstrated by a simple simulation as shown below. In this simulation, it is assumed that a shear sensitive cell will be damaged if the cell enters a high shear stress zone inside a certain part of the reactor (e.g., in the cavities of the circulation pump), where the shear stress level is beyond the threshold of the algae. Computational fluid dynamics (CFD) analyses on shear stress in centrifugal pumps show that the probability of cells entering regions inside the pump with shear stress levels that damage the cells depends on the residence time of the cells inside the pump, the rotational speed of the impeller, and the shape of the impeller (Song et al. 2003; Takiura et al. 1998; Zhou et al. 2003). The magnitude of the damage is therefore related to the proportion of the cells, *φ*, that pass this high shear stress zone per passage and the amount of passages (*n)* over time:

$$ \frac{N_{\mathrm{r}}}{N_0}={\left(1-\varphi \right)}^{\mathrm{n}} $$

where *N*_r_ is the cell concentration of the sensitive algae remaining viable and *N*_0_ is the cell concentration of sensitive algae before exposure. Figure 4 shows the percentage of intact cells remaining over time assuming that 1, 5, or 10 % of the cells are being damaged per passage.

The number of passages through the pump depends on the length of the tubular PBR and the flow velocity. At a pumping speed of 2 m^3^ h^−1^, the culture is recirculated 50 times per hour in the PBR. This means that the number of passes per day is about 1200. Even when only 1 % of the cells pass a high shear zone, it is clear that all sensitive cells would be inactivated within 1 day. However, not all cells might be shear stress sensitive. Most likely, the cells which are in the dividing stage are especially vulnerable to shear stress. Assuming that at a given time about 30 % of the microalgal cells are in the dividing stage (Coats and Heinbokel 1982) and that the generation time is about 1 day, the time that a cell is vulnerable to shear stress is about 7.2 h per day. In those 7.2 h, the number of passages is 360, which leads to an inactivation of 97 % of the sensitive cells. Only 3 % will remain for further growth, which is clearly too low to maintain or even increase the number of microalgae cells. This means that in the tubular PBR, almost all cells are expected to be inactivated within one or a few days, which is in line with the experimental observations.

### Design of closed photobioreactor systems for shear sensitive microalgae

Theoretically, two possible routes are possible to avoid cell damage due to shear stress. The first one is reducing or avoiding high shear stress zones in the reactor. This option, however, might be quite difficult to achieve. Lowering the flow rate too much will lead to other problems, because high turbulence is needed for keeping the microalgae suspended and the use of light and nutrients is enhanced by turbulent mixing (Richmond 2013). Another option is to reduce the number of passages. Many commercial tubular PBRs consist of longer tubes with a length of 100 m (Pulz et al. 2013) and with flow velocities applied in PBRs between 0.3 and 0.5 m s^−1^ (Norsker et al. 2011). This reduces the number of passages of the shear stress sensitive cells, which are in the dividing stage, to about 90 per day. In the case of 1 % of the cells being affected per pump passage in a commercial PBR, 60 % of the sensitive cells would still be damaged. It is obvious that more cells will be damaged, if a higher percentage of the cells is affected per passage through the pump (Fig. 4). Considering that the time needed for cell proliferation is about 1 day, the time is too short to overcome the damage done by high shear stress, even if the high shear stress region is small.

The tolerance to shear stress of various strains seems to be selective to the choice of recirculation pumps. Microalgae with rigid cell walls are shear stress tolerant, while species lacking a cell wall, coccolithophores with calcium carbonate containing coccospheres and diatoms with fragile siliceous frustules are sensitive to shear stress (Leupold et al. 2013; Moheimani et al. 2011; Vandanjon et al. 1999). *Phaeodactylum tricornutum* is an exception among the diatoms. Its cell wall structure does not contain siliceous valves but rather the cells hace a more rigid polysaccharide cell wall (Borowitzka and Volcani 1978; Tesson et al. 2009). It therefore can be successfully cultivated in tubular PBRs with centrifugal pumps, although some reduction of productivity has been reported at higher flow rates causing high shear zones in the reactor (Alías et al. 2004; Silva Benavides et al. 2013).

The fact that *I. galbana*, *S. costatum*, and a species of the genus *Chaetoceros* were reported to be successfully cultivated in tubular PBRs recirculated with airlift pumps (Molina Grima et al. 1994; Krichnavaruk et al. 2005; Loubière et al. 2009; Monkonsit et al. 2011; Van Bergeijk et al. 2010) suggests that hydrodynamic forces exerted in the tubes of PBRs were probably not high enough to negatively affect the growth. Airlift pumps, which are causing lower shear stress than centrifugal pumps (Carvalho et al. 2006), seem to be the best option for the recirculation of shear stress sensitive microalgae. Peristaltic pumps, eccentric rotor pumps, and diaphragm pumps may be interesting alternatives as well. These pumps have been shown to be more gentle than centrifugal or positive displacement rotary vane pumps (Jaouen et al. 1999). However, further research on the actual shear stress levels occurring in these pumps is recommended in order to find whether shear stress levels are sufficiently low to allow successful algae cultivation in PBRs.

## Conclusion

Four different microalgae species used as feed for shellfish in hatcheries were tested for their shear stress sensitivity. *T. suecica* was found to be the only shear stress tolerant species tested, which viability was not negatively affected by a maximum applied shear stress level of 88 Pa. *T. suecica* was also successfully grown in a tubular PBR driven by a centrifugal pump. *I. galbana*, *S. costatum*, and *C. muelleri* were not able to grow in the tubular PBR recirculated by a centrifugal pump at its lowest speed. Shear stress levels between 1.2 and 5.4 Pa caused a reduction in viability of the shear stress sensitive species *I. galbana*, *S. costatum*, and *C. muelleri*. In order to increase the feasibility of the production of microalgae for aquaculture in fully-automated PBRs, high shear stress zones in the reactor (including the pump) should be avoided when designing a culture system for shear stress sensitive microalgal species.
